# Down-regulation of ghrelin receptors on dopaminergic neurons in the substantia nigra contributes to Parkinson’s disease-like motor dysfunction

**DOI:** 10.1186/s13041-018-0349-8

**Published:** 2018-02-20

**Authors:** Yukari Suda, Naoko Kuzumaki, Takefumi Sone, Michiko Narita, Kenichi Tanaka, Yusuke Hamada, Chizuru Iwasawa, Masahiro Shibasaki, Aya Maekawa, Miri Matsuo, Wado Akamatsu, Nobutaka Hattori, Hideyuki Okano, Minoru Narita

**Affiliations:** 1 Department of Pharmacology, Hoshi University School of Pharmacy and Pharmaceutical Sciences, Ebara, Shinagawa-ku, Tokyo, 142-8501 Japan; 2 Department of Physiology, Keio University School of Medicine, 35 Shinanomachi, Shinjuku-ku, Tokyo, 160-8582 Japan; 3 Laboratory of Molecular Genetics, The Institute of Medical Science, The University of Tokyo, 4-6-1 Shirokanedai, Minato-ku, Tokyo, 108-8639 Japan; 4 Center for Genomic and Regenerative Medicine, Juntendo University, School of Medicine, Bunkyo-ku, Tokyo, 113-8431 Japan; 5 Department of Neurology, Juntendo University School of Medicine, Bunkyo, Tokyo, 113-8421 Japan; 6 Life Science Tokyo Advanced Research Center (L-StaR), Hoshi University School of Pharmacy and Pharmaceutical Sciences, Ebara, Shinagawa-ku, Tokyo, 142-8501 Japan

**Keywords:** Parkinson’s disease, Ghrelin, GHSR, iPS, Dopamine neuron

## Abstract

Ghrelin exerts a wide range of physiological actions throughout the body and appears to be a promising target for disease therapy. Endogenous ghrelin receptors (GHSRs) are present in extrahypothalamic sites including the substantia nigra pars compacta (SNc), which is related to phenotypic dysregulation or frank degeneration in Parkinson’s disease (PD). Here we found a dramatic decrease in the expression of GHSR in PD-specific induced pluripotent stem cell (iPSC)-derived dopaminergic (DAnergic) neurons generated from patients carrying parkin gene (PARK2) mutations compared to those from healthy controls. Consistently, a significant decrease in the expression of GHSR was found in DAnergic neurons of isogenic PARK2-iPSC lines that mimicked loss of function of the PARK2 gene through CRISPR Cas9 technology. Furthermore, either intracerebroventricular injection or microinjection into the SNc of the selective GHSR1a antagonist [D-Lys3]-GHRP6 in normal mice produced cataleptic behaviors related to dysfunction of motor coordination. These findings suggest that the down-regulation of GHSRs in SNc-DA neurons induced the initial dysfunction of DA neurons, leading to extrapyramidal disorder under PD.

**Electronic supplementary material:**

The online version of this article (10.1186/s13041-018-0349-8) contains supplementary material, which is available to authorized users.

## Introduction

Parkinson’s disease (PD) is a common, debilitating, neurodegenerative disorder that is associated with progressive motor dysfunction. PD is characterized by the progressive loss of dopamine (DA) neurons, and the DA neurons that degenerate in PD primarily project to the substantia nigra pars compacta (SNc). The motor symptoms of PD manifest only after a significant loss of striatal (70–80%) DA concentration in the brain and are seen relatively late in disease progression. PARKIN (PARK2), an E3 ubiquitin ligase, is the most frequently mutated gene that has been causally linked to autosomal recessive early-onset familial Parkinson’s disease [1, 2]. Abnormalities of PARK2 have also been described in sporadic PD [3].

Induced pluripotent stem cells (iPSCs) give rise to all cells in an organism. A potential solution to the difficulty of modeling PD is to use reprogramming technology to generate disease-specific iPSCs. PD-specific iPSCs-derived DA neurons could recapitulate the pathological features of PD. The exact mechanism by which PARK2 causes PD-like syndromes and why dopaminergic neurons are primarily affected by a ubiquitously expressed mutation remain unknown [4, 5]. A monogenic form of PD-specific iPSCs-derived DA neurons could provide important clues for elucidating the pathogenesis of PD.

Ghrelin, an endogenous ligand for growth hormone secretagogue receptor (GHSR), which is classified as a G-protein coupled receptor, is a 28-amino acid peptide that regulates growth hormone secretion, food intake, reward-seeking behavior and memory performance [6–9]. While it is mainly secreted from the stomach [8], small amounts are produced in the brain [10]. GHSR is expressed in various brain areas including the SNc, hypothalamus, ventral tegmental area and hippocampus, where ghrelin directly modulates neuronal activity [6, 7, 11, 12]. In the SNc, ghrelin electrically activates dopaminergic neurons, and increases the dopamine concentration in the striatum via the specific blockade of KCNQ channel function [13]. It has also been reported that ghrelin has a neuroprotective effect to prevent the greater loss of SNc dopaminergic neurons in a 1-methyl-4-phenyl-1,2,3,6-tetrahydropyridine (MPTP) model of PD [11].

In the present study, we evaluated the changes in GHSR expression in dopaminergic neurons derived from PD-specific iPS cells. Furthermore, we confirmed that the inhibition of GHSR in DA neurons of the substantia nigra, using the microinjection of a selective GHSR inhibitor into the SNc, leads to motor dysfunction.

## Methods

### Human iPS cells

For control lines, we used two human iPS cell lines: 201B7 iPSCs were purchased from RIKEN BRC and kindly provided by Dr. Shinya Yamanaka [14] at Kyoto University and WD39 iPSCs were established [15] at Keio University. For PD (PARK2) lines, the patient A (PA9 and PA22) and patient B (PB2 and PB20) iPSCs were established by Dr. Imaizumi [15]. All of the iPSCs were maintained on feeder cells in iPSC culture media, as described previously [15]. All of the experimental procedures for iPS cell production were approved by the Ethics Committee of Keio University School of Medicine. All of the experimental procedures for cell differentiation and analysis were approved by the respective Ethics Committees of Keio University School of Medicine (Approval Number: 20–16-28) and Hoshi University School of Medicine (Approval Number: 28–008).

### In vitro differentiation of human iPSCs (hiPSCs)

DA neuron differentiation from iPSCs was performed according to a previously reported protocol [16–18]. Neural induction was initiated through the inhibition of both BMP and TGFβ signaling using the small molecules Dorsomorphin (DM, Sigma-Aldrich, St. Louis, MO, USA) and SB431542 (SB, Tocris Bioscience, Bristol, UK). The small molecule CHIR99021 (CHIR, Stemgent, Lexington, MA, USA), a GSK3β inhibitor, was added to stimulate the canonical WNT signaling pathway. For neural induction from single hiPSCs, hiPSCs were incubated with TrypLE™ Select (Gibco, Life Technologies, CA, USA) for 5–10 min and dissociated into single cells by pipetting. Cells were plated into a T75 flask and cultured in KBM (KOHJINBIO, Saitama, Japan) supplemented with B27 (Gibco, Life Technologies), 20 ng/mL basic-FGF (bFGF, PeproTech. Inc., Rocky Hill, NJ, USA), 10 μM Y-27632 (Wako, Tokyo, Japan), 10 ng/mL hLIF (Millipore, Billerica, MA, USA), 1 μM Purmorphamine (Calbiochem, San Diego, CA, USA), 2 μM SB (Tocris Bioscience), 100 ng/ml CHIR, 100 ng/ml Sonic hedgehog (Shh, R&D Systems Inc., Minneapolis, MN, USA) and 100 ng/ml FGF8b (PeproTech) in 4% oxygen for 7 or 12 days. Neurospheres were repeatedly passaged by dissociation into single cells, and then cultured in the same manner. Neurospheres at passage 3 were typically used for analysis. For terminal differentiation, dissociated neurospheres were allowed to adhere to poly-L-ornithine (Sigma-Aldrich)- and fibronectin (Sigma-Aldrich)-coated coverslips and cultured in KBM (KOHJINBIO) containing B27 (Gibco, Life Technologies), 20 ng/mL brain-derived neurotrophic factor (BDNF, R&D Systems), 20 ng/mL glial cell-derived neurotrophic factor (GDNF, R&D Systems), 200 μM ascorbic acid (Sigma-Aldrich), and 500 μM dibutyryl-cAMP (Sigma-Aldrich) for 10 days.

### Immunocytochemical analysis

Cells were fixed with 4% paraformaldehyde (PFA) and then washed three times with PBS. After cells were incubated with blocking buffer (PBS containing 5% normal fetal bovine serum and 0.3% Triton X-100) for 1 h at room temperature, they were incubated overnight at 4 °C with primary antibodies diluted with blocking buffer. The details of the primary antibodies and the dilution conditions are listed in Additional file 1: Table S1. The cells were again washed three times with PBS and incubated with secondary antibodies conjugated with Alexa Fluor 488 or Alexa Fluor 546 for 1 h at room temperature. After cells were washed three times with PBS, samples were mounted on slides with DAPI-Fluoromount-G™ (SouthernBiotech, Birmingham, AL, USA). Fluorescence of immunolabeling was detected using a light microscope BZ-X710 (KEYENCE, Osaka, Japan) or IX-73 (Olympus, Tokyo, Japan) and photographed with a digital camera using BZ-X Analyze software (KEYENCE) or cellSens software (Olympus).

### qRT-PCR

Total RNA was isolated from cells using an RNeasy mini kit (QIAGEN, Hilden, Germany) with DNase I treatment, and cDNA was prepared by using a SuperScript® VILO™ cDNA Synthesis Kit (Invitrogen, Waltham, MA, USA). The qRT-PCR analysis was performed with Fast SYBR Green Master Mix (Thermo Fisher Scientific, Waltham, MA, USA) on a StepOne Plus™ System (Applied Biosystems Inc., Foster City, CA, USA). Values were normalized to β actin (ACTB). The primer sets used in these experiments are listed in Additional file 2: Table S2.

### Generation of *PARK2* gene knock-in/knock-out (PARK2-KIKO line) by CRISPR-Cas9

We previously generated CRISPR/Cas9-dependent *PARK2*-KIKO line using 201B7 as control iPSCs, to evaluate *parkin* loss of function on DA neurons-derived from iPSCs (Kuzumaki et al., in submission). In brief, a targeting donor DNA plasmid (pUC- 5’3’PARK2- PurTK) was used to disrupt exon 2 of *PARK2* gene by homologous recombination. The CSIV-U6-*PARK2* (Ex2)-sgRNA-L&R-EF-Csy4-2A-Cas9 was used as a house-made all-in-one vector. The 201B7 was suspended in Opti-MEM (Thermo Fisher) containing Y-27632, house-made all-in-one vector and targeting donor DNA vector plasmid. Electroporation of plasmid DNA was performed using a NEPA21 electroporator (Nepa Gene Co., Ichikawa, Japan). As shown in Additional file 3: Figure S1, PARK2-KIKO clone was identified by PCR method with the primers listed in Additional file 4: Table S3.

### Animals

The present study was conducted in accordance with the Guiding Principles for the Care and Use of Laboratory Animals, Hoshi University, as adopted by the Committee on Animal Research of Hoshi University, which is accredited by the Animal Research Committee of Hoshi University. Male C57BL/6 J mice (Jackson Laboratory) were used in this study. All mice were housed at up to 6 mice per cage and kept in a temperature-controlled room (24 ± 1 °C) maintained on a 12 h light-dark cycle (light on at 8 a.m.). Food and water were available ad libitum.

### Drugs

[D-Lys-3]-GHRP-6 (Tocris, Bristol, United Kingdom) and morphine hydrochloride (Daiichi-Sankyo Co., Ltd., Tokyo, Japan) were used in this study.

### Intracerebroventricular administration

Intracerebroventricular (i.c.v.) administration was performed according to the method described previously [19]. A 2 mm double needle (Natsume Seisakusho) attached to a 25 μl Hamilton microsyringe was inserted into the unilateral injection site using a V-shaped holder to hold the head of the mouse. On the day of the assay, [D-Lys-3]-GHRP-6 (0.3 to 10 nmol/ mouse) was injected into the hole. The injection volume was 4 μl for each mouse.

### Cannula implantation into the SNc

Stereotaxic injections were performed under isoflurane (3%) anesthesia and using small-animal stereotaxic instruments (RWD Life Science, Shenzhen, China). Mice were placed in a stereotaxic apparatus and the skull was exposed. A small hole was then made in the skull using a dental drill. A guide cannula (EIM-54; Eicom, San Diego, CA, USA) was implanted into the SNc (from bregma: AP -3.0 mm, ML ±1.2 mm, DV -4.3 mm). [D-Lys-3]-GHRP-6 (1 to 5 nmol/side) was microinjected at a rate of 0.25 μl min^− 1^ for 4 min. At the end of injection, the injection cannula was kept in the SNc for an additional 2 min before removal and then replaced by a stylet.

### Rotarod assay test

Motor coordination was assessed using the rotarod test. Mice were individually placed on a slowly rotating rod (4 rpm/min), and subjected to continuous acceleration at 20 rpm/min; the time at which the mouse fell off the rod was recorded. The test was performed 10 min after i.c.v. injection of either saline vehicle or [D-Lys-3]-GHRP-6 (0.3 to 10 nmol/mouse), or 15 min after microinjection of either saline vehicle or [D-Lys-3]-GHRP-6 (1 to 5 nmol/side).

### Balance beam test

The apparatus consisted of a 1 m–long bar (28 or 11 mm in diameter) with a black escape box on one end (O’HARA & Co., LTD., Tokyo, Japan). Mice were acclimated to enter the escape box on the 28 mm-diameter bar for 2 days before testing. The latency to reach the box on the 11 mm-diameter bar was measured  (cut off time = 60s). The test was performed 10 min after microinjection of either saline vehicle or [D-Lys-3]-GHRP-6 (1 to 5 nmol/side).

### Locomotor assay

After 30 min of habituation to the apparatus, the locomotor activity of mice was measured by a Three-point Meter (O’HARA & Co., LTD). With this device, the position of the mouse is detected when the infrared beams positioned along the X and Y axes around the cage are interrupted. This device detects the movement of the whole body of the target animal, without being misled by the movement of the tail or any other part of the mouse. Counts of hyperlocomotor activity were obtained at 1-min intervals for 120 min after the injection of morphine hydrochloride (Daiichi-Sankyo Co., Ltd., Tokyo, Japan).

### Statistics

The data are presented as the mean ± S.E.M. The statistical significance of differences between groups was assessed by an unpaired t-test or one-way analysis of variance (ANOVA) test followed by the Bonferroni’s multiple comparison test. All statistical analyses were performed with GraphPad Prism (GraphPad Software, La Jolla, CA, USA). A *p* value of < 0.05 was considered to reflect significance.

## Results

### Differentiation of *PARK2*-specific iPSCs into DA neurons

**Fig. 1:**
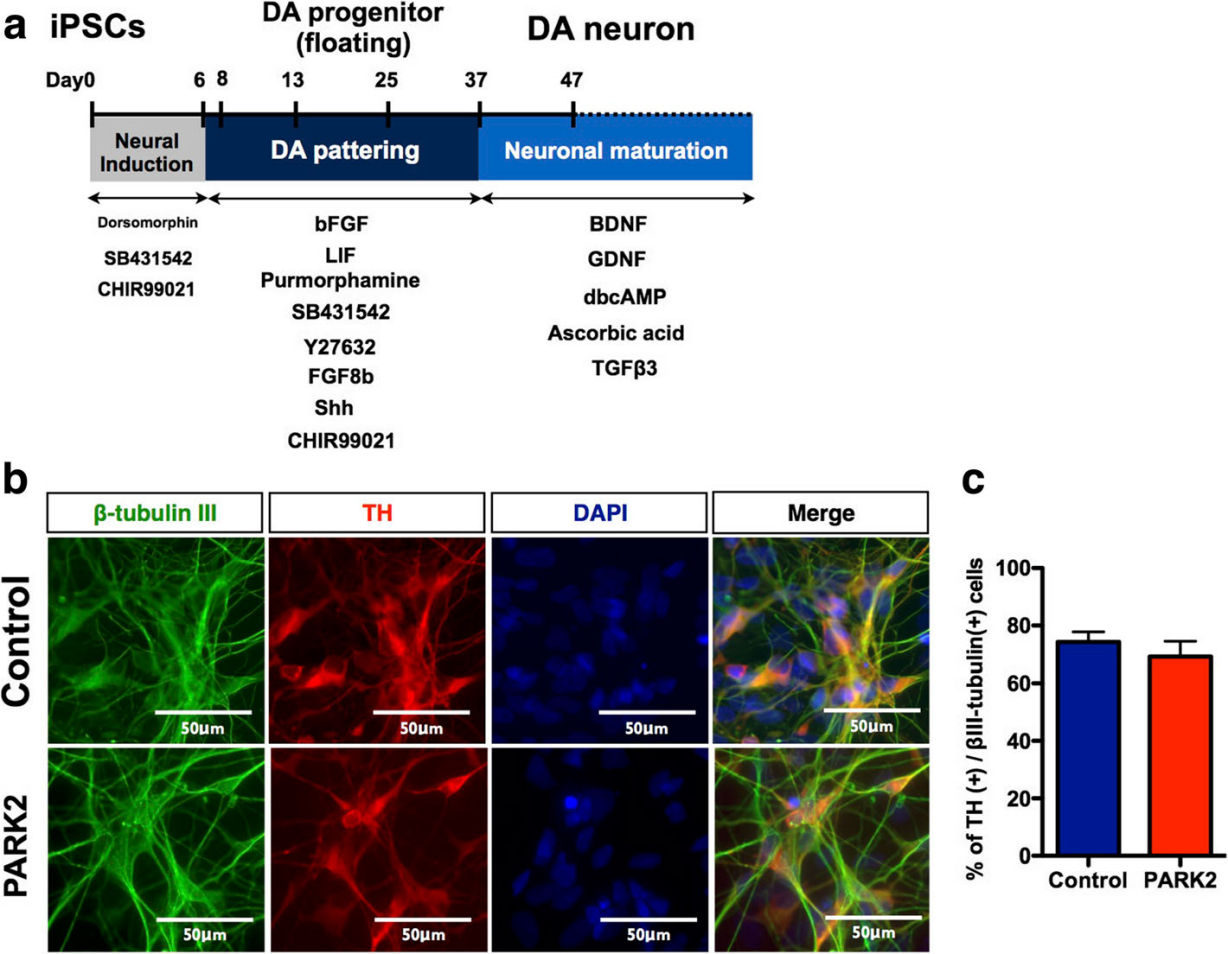
Ghrelin receptor (GHSR) expression in dopaminergic neurons derived from control and *PARK2*-specific iPSCs. **a** Schematic of the induction of a DA-enriched culture protocol. **b** Double-labeling for the dopaminergic neuron marker tyrosine hydroxylase (TH, red) and neurons (βIII-tubulin, green) of control and *PARK2*-specific iPSC-derived dopaminergic neurons. Scale bar = 50 μm. **c** Quantitative data of the percentage of TH positive cells per βIII-tubulin positive cells

Since PD is defined pathologically by the progressive degeneration of DA neurons, we modified the method used to generate a DA neuron-enriched culture by treating iPSC-derived cells with several small molecules that lead to the formation of ventral midbrain cells, including DA neurons (Fig. 1a). Most of the differentiated cells derived from control and *PARK2*-specific iPSCs were labeled by antibodies to βIII-tubulin (a neuron-specific marker) and TH (a dopaminergic neuronal marker) (Fig. 1b). There was no significant difference in the ratio of TH-positive DA neurons between control and PARK2 iPSCs-derived neurons (Fig. 1c). In the previous study, we demonstrated that this iPSC-based model of PARK2 recapitulated the vulnerability of DA neurons [16, 20].

### Decreased expression of GHSR in *PARK2*-specific iPSC-derived DA neurons

**Fig. 2:**
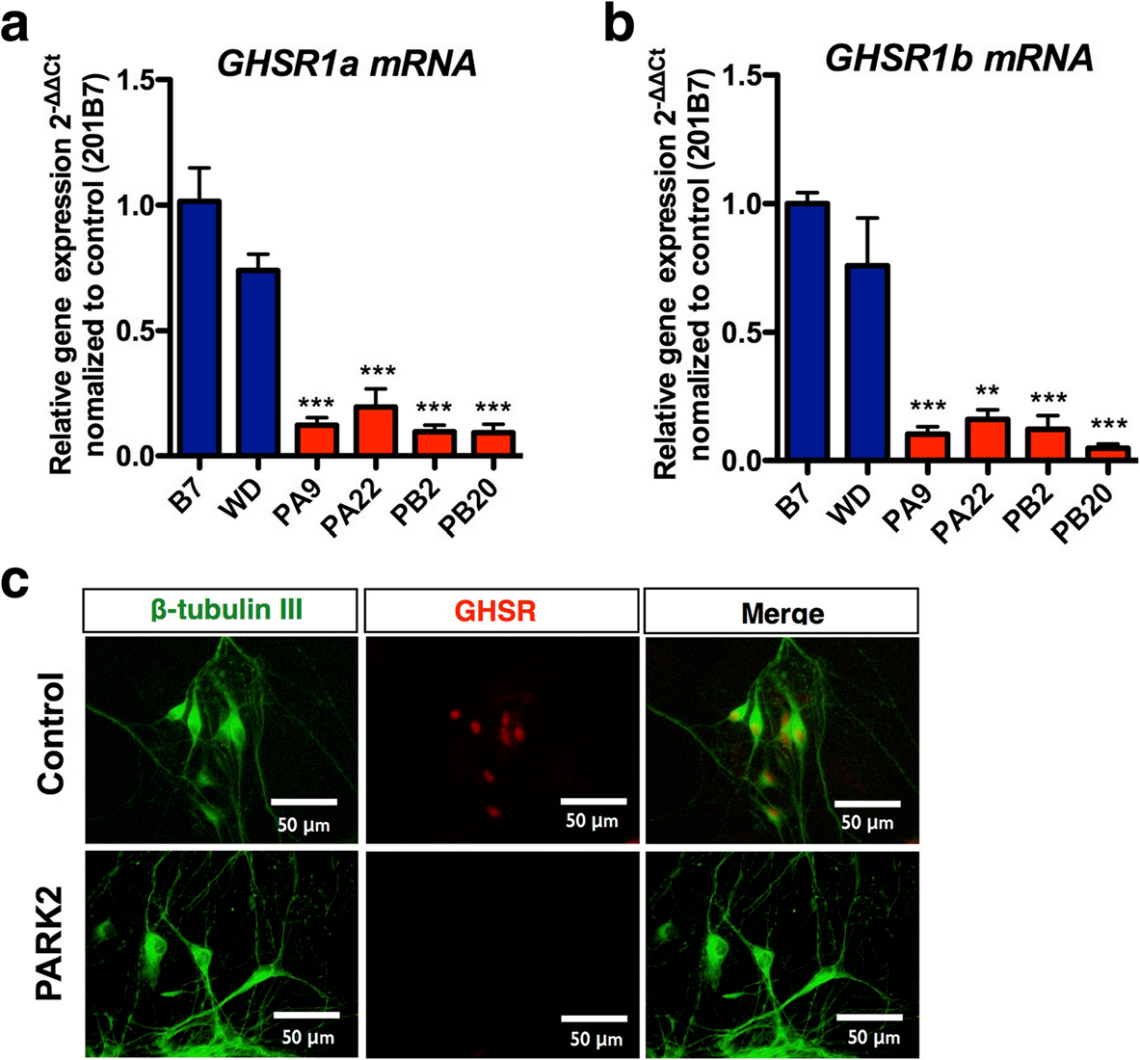
Ghrelin receptor (GHSR) expression in dopaminergic neurons derived from control and *PARK2*-specific iPSCs. **a-b** mRNA expression of GHSR1a (**a**) and GHSR1b (**b**) between control and Parkinson’s disease-specific iPS cells derived-dopaminergic neurons. **p < 0.01, ***p < 0.001 vs. control iPS cell-derived dopaminergic neurons. **c** Immunocytochemical analysis for TH (green) and ghrelin receptor (red) in control and *PARK2* iPS cell-derived dopaminergic neurons

We observed a significant decrease in mRNA levels of *GHSR1a* and *GHSR1b* in *PARK2-*specific iPSC-derived DA neurons (Fig. 2a-b, **p < 0.01, ***p < 0.001 vs. control-iPSC derived DA neurons). Furthermore, the protein levels of GHSR were mostly abolished in DA neurons-derived from *PARK2-*specific iPSCs (Fig. 2c).

### Recapitulation of *GHSR* expression in isogenic *PARK2*-KIKO iPSC-derived DA neurons

**Fig. 3:**
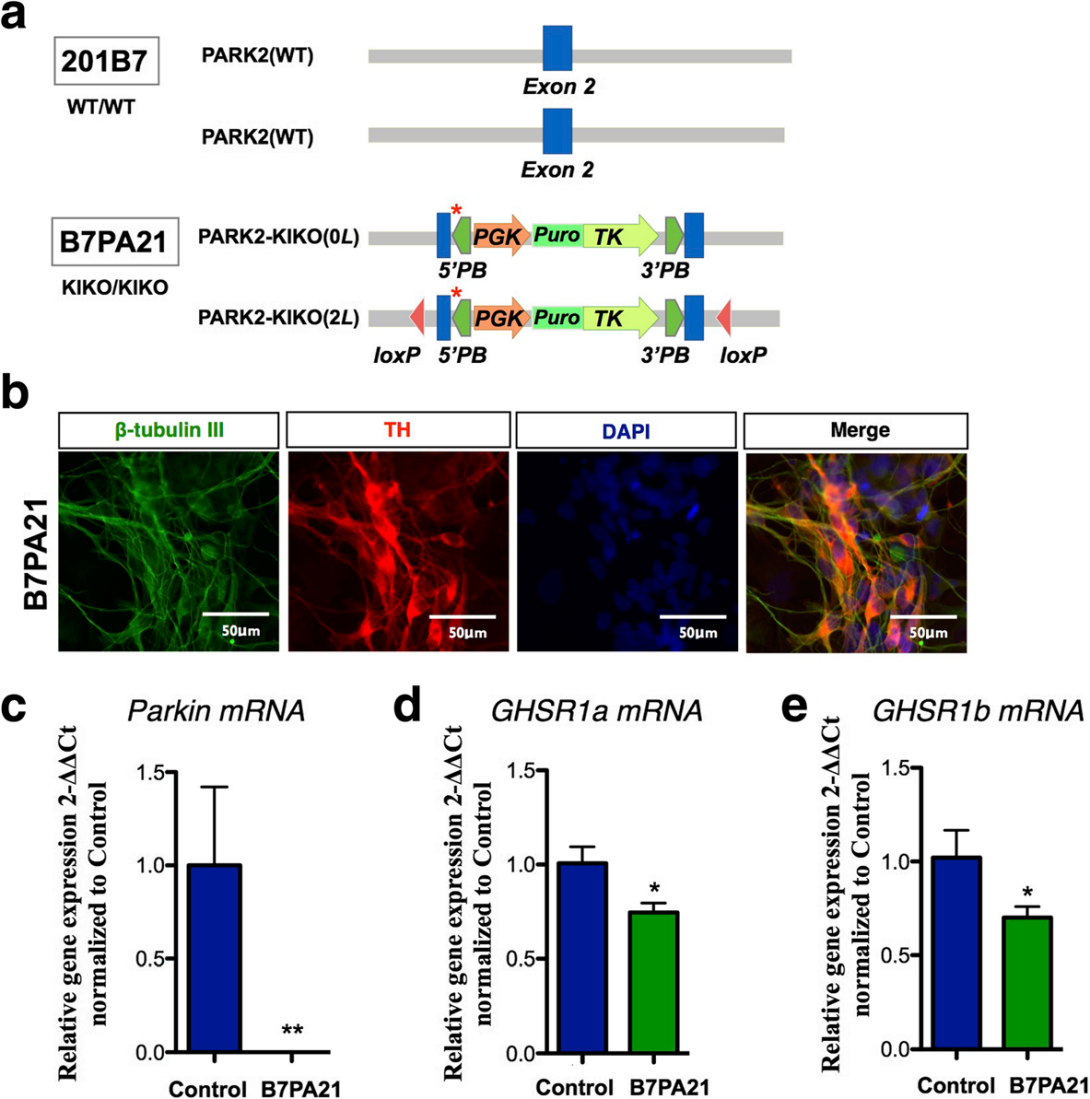
Decreased expression of GHSR in isogenic *PARK2*-KIKO iPSC-derived DA neurons. **a** Generation of isogenic *PARK2*-KIKO iPSCs. Schematic illustration of the gene-editing strategy for knock-in of the stop codon and the puromycin resistance gene into control iPSCs (201B7). **b** Double-labeling for the DA neuron marker tyrosine hydroxylase (TH, red) and the neuronal marker β-tubulin III (TUJ1, green) of control and *PARK2*-KIKO iPSC-derived dopaminergic neurons. **c** Expression level of Parkin mRNA in differentiated DA neurons derived from the control and *PARK2*^*−*^KIKO iPSC groups. **p < 0.01 vs. control. **d-e** The expression levels of GHSR1a (**d**) and GHSR1b (**e**) in differentiated dopaminergic neurons derived from the control and *PARK2*-KIKO iPSC groups. *p < 0.05 vs. control

We next used isogenic iPSC lines mimicking loss of function of the *PARK2* gene through CRISPR Cas9 technology in the healthy control iPSC line 201B7 (Fig. 3a). We found that one of the *PARK2*-KIKO iPSC lines, B7PA21 differentiated to TH-positive neurons (Fig. 3b). We confirmed the knock-down of Parkin mRNA in DA neurons-derived from *PARK2-*KIKO isogenic iPSCs (Fig. 3c). Under these conditions, we found a significant decrease in the mRNA expression of GHSR1a and GHSR1b in the *PARK2*-KIKO isogenic line, similar to that seen in the familial *PARK2* lines (Fig. 3d-e).

### Effects of intracerebroventricular injection of the selective GHSR1a antagonist [D-Lys3]-GHRP-6 on motor coordination

**Fig. 4:**
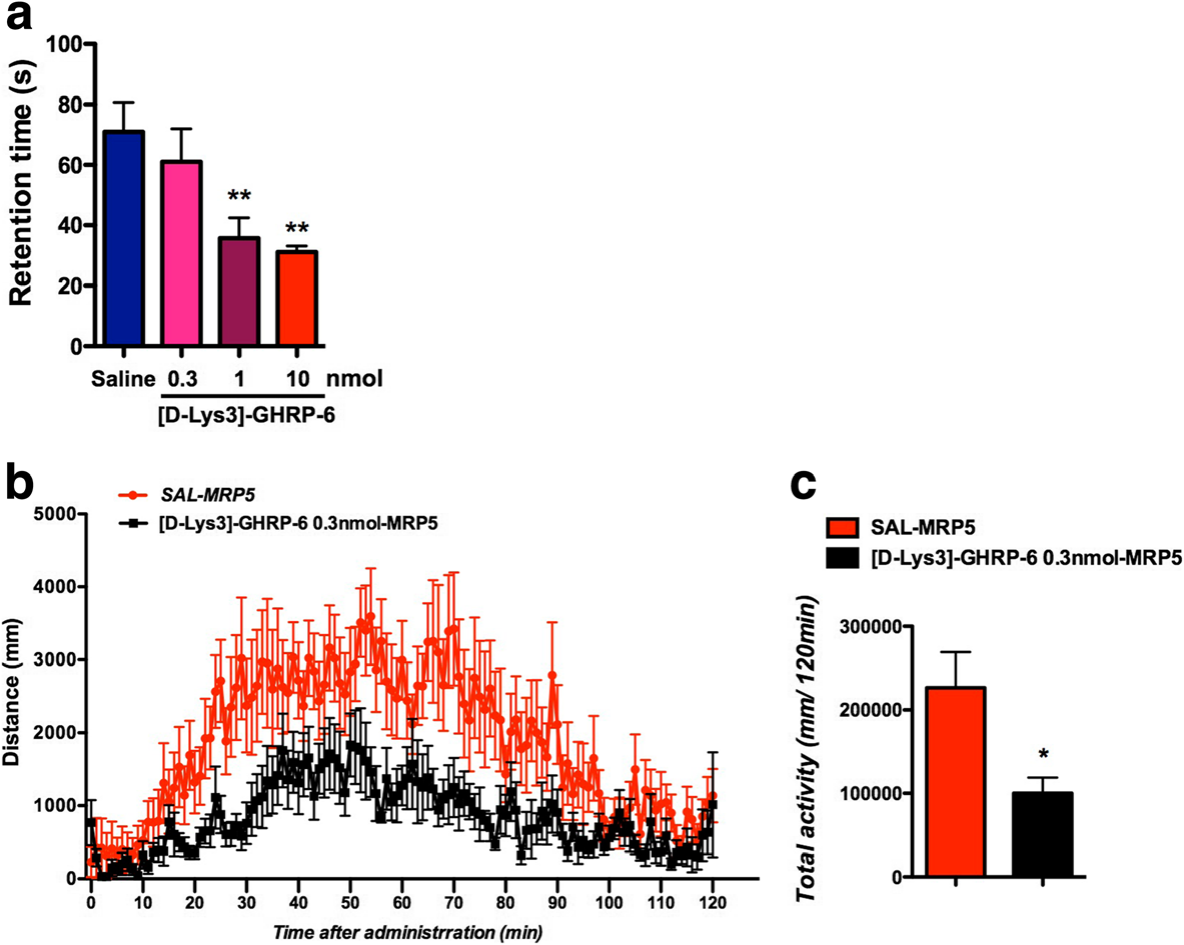
Effects of intracerebroventricular injection of the selective GHSR1a antagonist [D-Lys3]-GHRP-6 on motor coordination. **a** Average latency to fall in the rotarod test. ^**^p < 0.01 vs. SAL. **b** Time-course change in the locomotor-enhancing effect of morphine (5 mg/kg, s.c.) after treatment with [D-Lys3]-GHRP-6 at 0.3 nmol (*n* = 7) or saline (*n* = 8). Each point represents the mean activity distance for 1 min with SEM. **c** Total locomotor activity induced by morphine (5 mg/kg, s.c.) after treatment with [D-Lys3]-GHRP-6 at 0.3 nmol (n = 7) or saline (n = 8). Each column represents the mean total activity distance for 120 min with SEM. ^*^p < 0.05 vs. SAL-MRP5

To evaluate the in vivo effect of the blockade of central GHSR1a, normal mice were subjected to intracerebroventricular (i.c.v.) injection of the selective GHSR1a antagonist [D-Lys3]-GHRP-6 (0.3 to 10 nmol/mouse). The i.c.v. injection of [D-Lys3]-GHRP-6 in mice induced a dose-dependent impairment of motor coordination based on the rota-rod performance test (Fig. 4a). [D-Lys3]-GHRP-6 given i.c.v. at 0.3 nmol, which alone had no effect on motor coordination, significantly inhibited morphine-induced hyperlocomotion (Fig. 4b-c, *p < 0.05 vs. SAL-MRP5).

### Effects of intra-SNc injection of [D-Lys3]-GHRP-6 on motor coordination

**Fig. 5:**
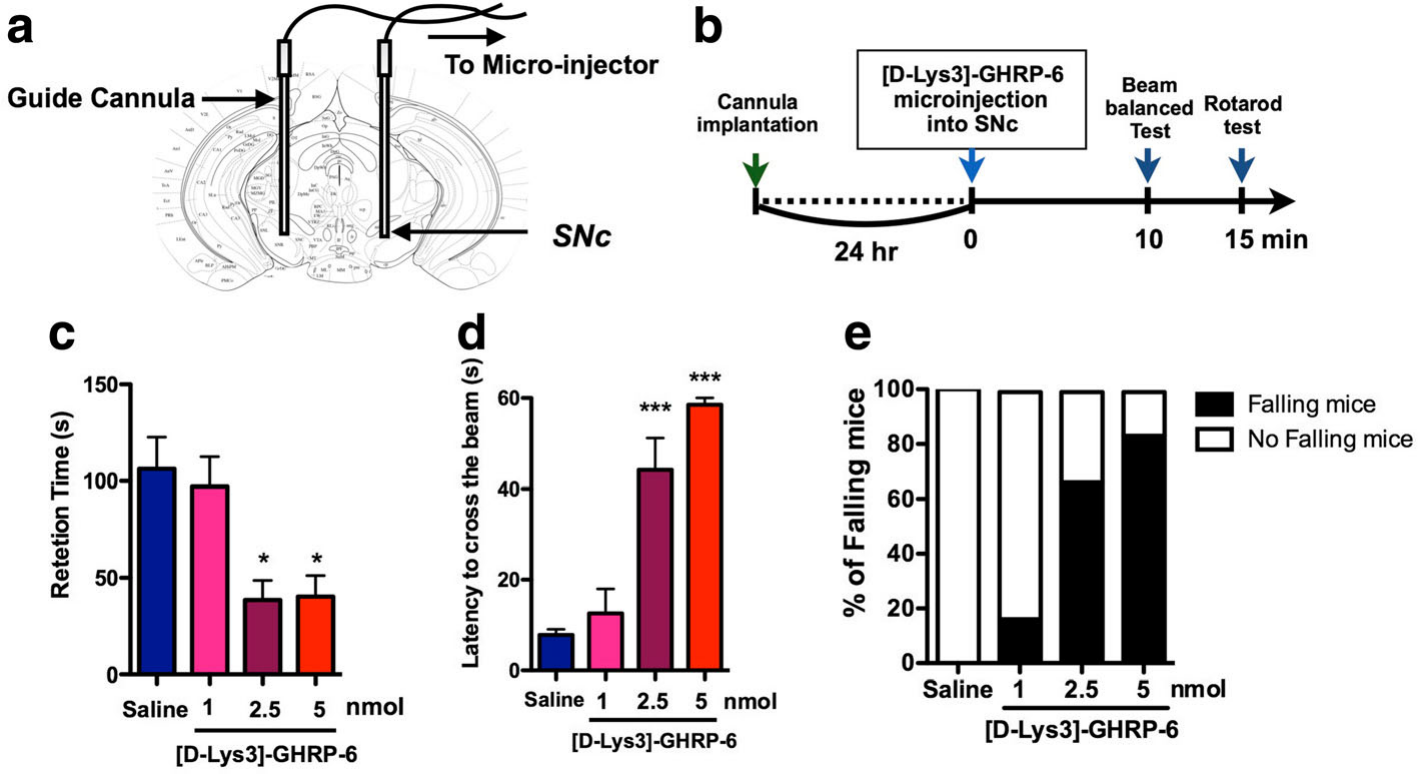
Effects of intra-SNc injection of [D-Lys3]-GHRP-6 on motor coordination. **a** Microinjection sites of [D-Lys3]-GHRP-6 in the SNc. Plates show coronal sections of the mouse brain. **b** Schedule for the experiment. **c** Accelerated rotarod test (4-20 rpm). The line graph shows the average latency to fall in the rotarod test for 15 min after bilateral microinjection of [D-Lys3]-GHRP-6 (1, 2.5 or 5 nmol / each site) or saline (*n* = 6/group) into the SNc. ^*^p < 0.05 vs. Saline. **d-e** The balance beam test was performed 10 min after the microinjection of [D-Lys3]-GHRP-6 (1, 2.5 or 5 nmol / each site) or saline (n = 6/group) into the SNc. ***p < 0.001 vs. Saline

To evaluate the in vivo effect of the blockade of GHSR1a, a guide cannula was implanted into the SNc for microinjection (Fig. 5a). One day after cannula implantation (Fig. 5b), microinjection of [D-Lys3]-GHRP-6 (1 to 5 nmol/side) into the SNc of normal mice produced a significant and dose-dependent impairment of motor coordination in the rota-rod test (Fig. 5c, *p < 0.05 vs. saline). In the balance beam test to further evaluate catalepsy behaviors, microinjection of [D-Lys3]-GHRP-6 into the SNc significantly increased the latency to cross the beam and increased the number of mice that fell from the beam (Fig. 5d-e, ***p < 0.001 vs. saline). These results suggest that the blockade of GHSR activation on DA neurons of the SNc induced motor dysfunction.

## Discussion

The identification of cell biological or biochemical changes in the initial stages of Parkinson’s disease, before the onset of symptoms, has been difficult through the use of analyses conducted on postmortem brains. With the recent development of iPS cell technologies, it has become possible to establish pluripotent stem cells from the somatic cells of anyone, regardless of race, genetic background, or the presence of disease symptoms. We have established iPS cells from cutaneous fibroblasts obtained from patients with the PARK2 form of familial PD (Patient A: female with an exon 2–4 deletion mutation; Patient B: male with an exon 6–7 deletion mutation) by performing retroviral gene transduction (*Oct4*, *Sox2, Klf4*, and *c-Myc*) [15, 21]. In the previous study, we demonstrated that this iPSC-based model of PARK2 recapitulated the vulnerability of DA neurons with a significant increase in ROS production [16, 20]. In our preliminary DNA microarray study, a dramatic decrease in mRNA levels of *GHSR1a* and *GHSR1b* was found in *PARK2-*specific iPSC-derived DA neurons (unpublished observation). In the present q-PCR assay, we confirmed that a significant decrease in both mRNAs was detected compared to a control in *PARK2-*specific iPSC-derived DA neurons. Furthermore, the level of GHSR protein was clearly down-regulated in DA neurons in *PARK2-*specific iPSC-derived DA neurons. To evaluate whether *PARK2* mutation is sufficient to cause the observed phenotype, we generated isogenic iPSC lines mimicking loss of function of the *PARK2* gene in a healthy control iPSC line. Under these conditions, we consistently found the significant, but not dramatic, decrease in the mRNA expression of both GHSR1a and GHSR1b in the *PARK2*-KIKO isogenic line. Although further analyses will be required to identify how PARK2 mutation could affect the expression of GHSR1a, PARK2 mutation may lead to the possible changes in the transfer of ubiquitin onto substrate proteins, which could affect the transcriptional level of GHSR1a. We thus hypothesized that although the deletion of *PARK2* gene would, at least in part, contribute to the decrease in the mRNA expression of both GHSR1a and GHSR1b, *PARK2-*specific iPSC-derived DA neurons from patients could be influenced by another genomic mutant factors to induce the dramatic knockdown of both mRNAs.

GHSR1a is the only functional ghrelin receptor that has been characterized to date. It is a G protein-coupled 7-transmembrane receptor that was first cloned from the pituitary and hypothalamus [22]. It has been reported that GHSR1a is localized in dopaminergic neurons of the SNc [23]. Higher numbers of TH and GHSR co-expressing neurons have been identified within the substantia nigra pars compacta [23]. Ghrelin has been shown to have neuroprotective effects in numerous animal models of neurological disorders, including PD. Studies using the mitochondrial toxin MPTP, which selectively kills dopaminergic neurons in the SNc, have shown that i.p. injection of ghrelin restricts dopamine cell loss in the SNc and the loss of dopamine in the striatum in mice [11, 24, 25]. Ghrelin activates SNc dopaminergic neurons, increases the expression of tyrosine hydroxylase (which is involved in the biosynthesis of dopamine) in the midbrain, and increases dopamine turnover in the dorsal striatum [11]. In the present study, we investigated whether the direct inhibition of GHSR function in the brain including the SNc could affect motor coordination. Either i.c.v. or intra-SNc injection of the selective GHSR1a antagonist [D-Lys3]-GHRP-6 in normal mice induced dose-dependent cataleptic behaviors related to the dysfunction of motor coordination. Furthermore, [D-Lys3]-GHRP-6, given i.c.v. at a dose which alone had no effect on motor coordination, caused a significant inhibition of the dopamine-related hyperlocomotion induced by the systemic administration of morphine. These findings suggest that deficits in GHSR activity in SNc-dopamine neurons could cause marked motor impairment. Inconsistently, it has been reported that GHSR^−/−^ mice did not show reduced performance in the rotarod test [26]. One reason for this discrepancy may be the possibility of significant compensation in the dopaminergic system when genes are deleted or overexpressed in the germline and during development of the dopaminergic system. In fact, germline deletion of parkin fails to lead to the loss of dopaminergic neurons, whereas adult conditional knockout of parkin leads to a progressive loss of dopamine neurons [27, 28]. Thus, it is likely that a conditional knockout technique in adults will be required to evaluate DA-related behaviors under PD.

In conclusion, we found that the expression level of GHSR was dramatically decreased in DA neurons under PD using disease-specific iPSCs. Furthermore, treatment by the injection of a selective GHSR1a inhibitor into the SNc of normal mice induced Parkinson’s disease-like behaviors. Taken together, these results indicate that the down-regulation of GHSR in DA neurons may correspond to the initial dysfunction of DA neurons, leading to extrapyramidal disorder under PD.

## Additional files

Additional file 1: Table S1. List of antibodies used for immunocytochemical analysis. (JPEG 244 kb)

Additional file 2: Table S2. List of primers used for qRT-PCR analysis. (JPEG 308 kb)

Additional file 3: Figure S1. Detection of a selection marker cassette knock-in by PCR. Genotyping by PCR was performed by using the primers listed in Table S3. For the detection of alleles of wildtype or indels, 5’PARK2-PCR-Fw and 3’PARK2-PCR-Rv, PARK2-Exon2-PCR-Fw and PARK2-Exon2-PCR-Rv were used. A large 5082-bp and 3464-bp fragment derived from knock-in allele is also detectable in this primer set. For the detection of alleles of knock-in, 5’PARK2-PCR-Fw and PGKp-Rv for detection of the 5′ knock-in fragment, and PuroR-Fw and 3’PARK2-PCR-Rv for detection of the 3’knock-in fragment were used. (JPEG 429 kb)

Additional file 4: Table S3. List of primers used for PCR analysis. (JPEG 291 kb)

## Abbreviations

BDNF: Brain-derived neurotrophic factor
DA: Dopamine
DAnergic: dopaminergic
DM: Dorsomorphin
GDNF: Glial cell-derived neurotrophic factor
GHSR: Growth hormone secretagogue receptor
iPSC: Induced pluripotent stem cell
PD: Parkinson’s disease
PFA: Paraformaldehyde
SNc: Substantia nigra pars compacta
