# Supplementary figures and images for: Down-regulation of ghrelin receptors on dopaminergic neurons in the substantia nigra contributes to Parkinson’s disease-like motor dysfunction
Source: Mol Brain. 2018 Feb 20;11:6. doi: 10.1186/s13041-018-0349-8 (PMC5819262; doi:10.1186/s13041-018-0349-8)

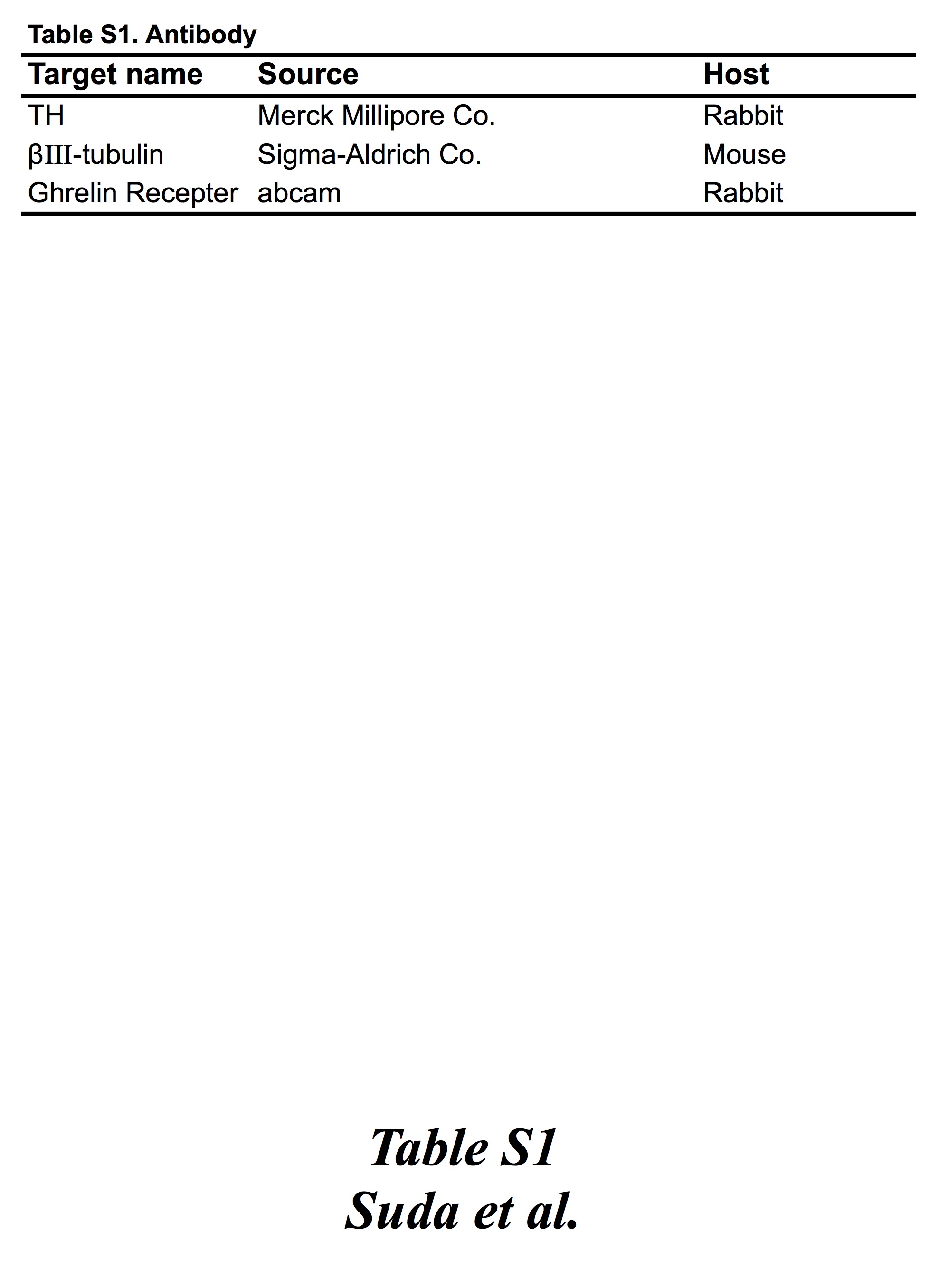

Supplement: Supplementary file 1 — List of antibodies used for immunocytochemical analysis. (JPEG 244 kb) [file 13041_2018_349_MOESM1_ESM.jpg]

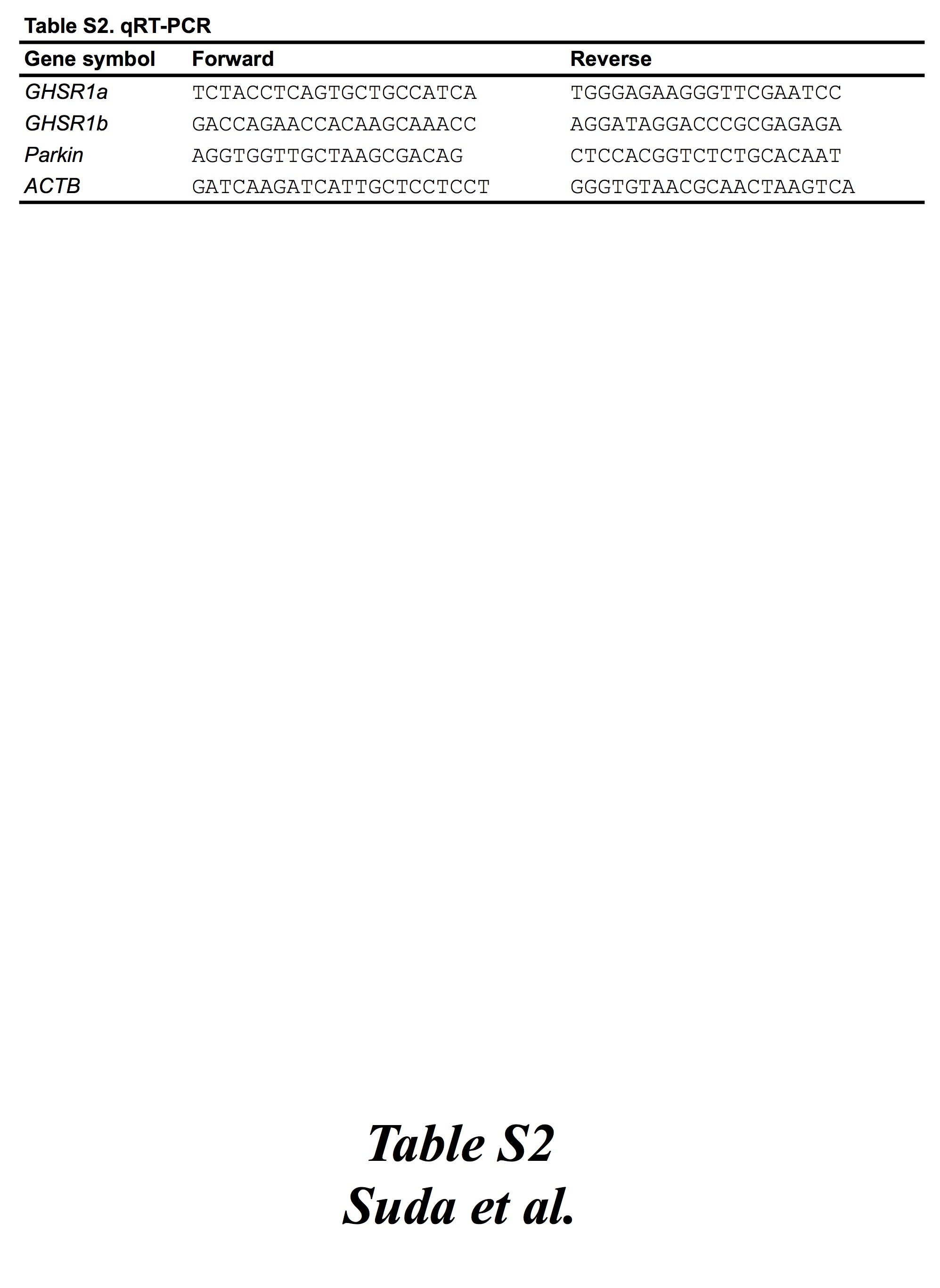

Supplement: Supplementary file 2 — List of primers used for qRT-PCR analysis. (JPEG 308 kb) [file 13041_2018_349_MOESM2_ESM.jpg]

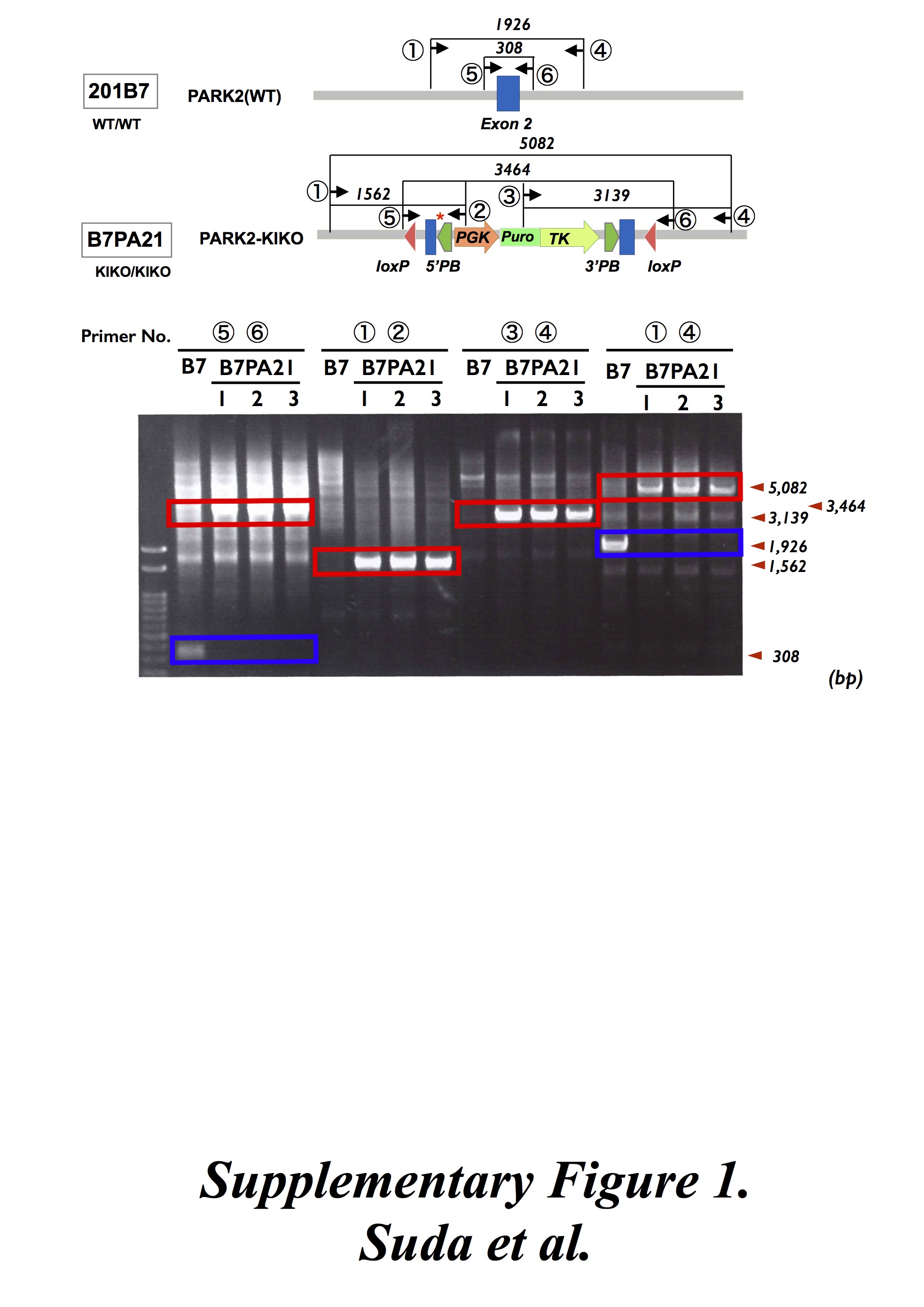

Supplement: Supplementary file 3 — Detection of a selection marker cassette knock-in by PCR. Genotyping by PCR was performed by using the primers listed in Table S3. For the detection of alleles of wildtype or indels, 5’PARK2-PCR-Fw and 3’PARK2-PCR-Rv, PARK2-Exon2-PCR-Fw and PARK2-Exon2-PCR-Rv were used. A large 5082-bp and 3464-bp fragment derived from knock-in allele is also detectable in this primer set. For the detection of alleles of knock-in, 5’PARK2-PCR-Fw and PGKp-Rv for detection of the 5′ knock-in fragment, and PuroR-Fw and 3’PARK2-PCR-Rv for detection of the 3’knock-in fragment were used. (JPEG 429 kb) [file 13041_2018_349_MOESM3_ESM.jpg]

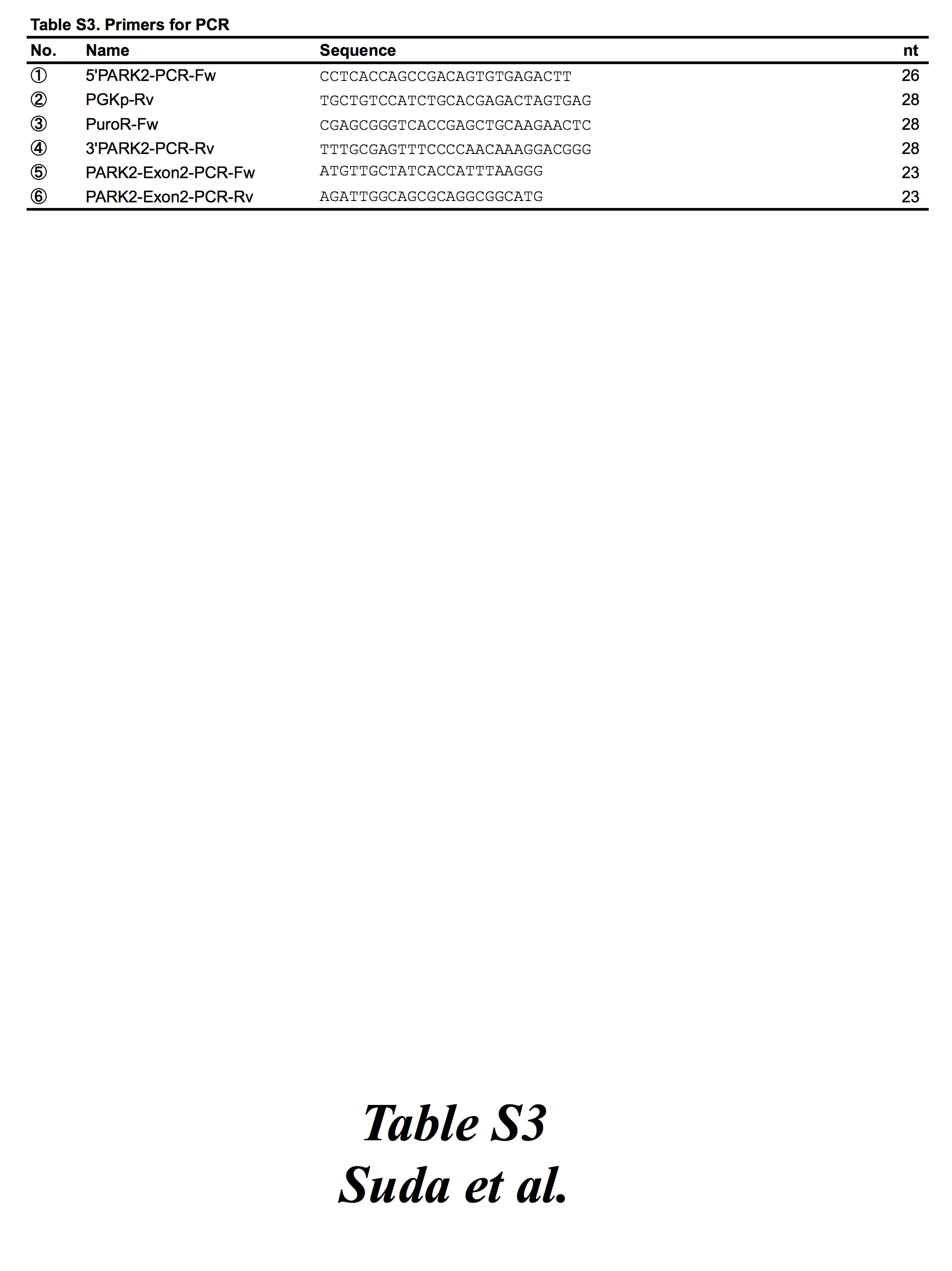

Supplement: Supplementary file 4 — List of primers used for PCR analysis. (JPEG 291 kb) [file 13041_2018_349_MOESM4_ESM.jpg]
